# Preoperative computed tomography-guided localization for multiple pulmonary nodules: comparison of methylene blue and coil

**DOI:** 10.1186/s13019-022-01941-4

**Published:** 2022-08-19

**Authors:** Sheng-Feng Zhang, Hai-Ri Liu, Ai-Li Ma, Er-Liang Li

**Affiliations:** 1 Department of Radiology, The Fourth People’s Hospital of Taizhou, Taizhou, China; 2 Department of Radiology, Xuzhou Central Hospital, 199 Jiefang Road, Xuzhou, China

**Keywords:** Computed tomography, Coil, Methylene blue, Multiple pulmonary nodules

## Abstract

**Background:**

Preoperative computed tomography (CT)-guided localization has been used to guide the video-assisted thoracoscopic surgery (VATS) sublobar (wedge or segmental) resection for pulmonary nodules (PNs). We aimed to assess the relative efficacy and safety of CT-guided methylene blue (MB)- and coil-based approaches to the preoperative localization of multiple PNs (MPNs).

**Methods:**

Between January 2015 and December 2020, 31 total cases suffering from MPNs at our hospital underwent CT-guided localization and subsequent VATS resection in our hospital, of whom 15 and 16 respectively underwent MB localization (MBL) and coil localization (CL). The clinical effectiveness and complication rates were compared between 2 groups.

**Results:**

The PN- and patient-based technical success rates in the MBL group were both 100%, whereas in the CL group they were 97.2% (35/36) and 93.8% (15/16), respectively, with no substantial discrepancies between groups. Patients in the MBL group illustrated a substantially shorter CT-guided localization duration compared with the CL group (18 min vs. 29.5 min, *P* < 0.001). Pneumothorax rates (*P* = 1.000) and lung hemorrhage (*P* = 1.000) were comparable in both groups. In the MBL and CL groups, the median interval between localization and VATS was 1 h and 15.5 h, respectively (*P* < 0.001). One-stage VATS sublobar resection of the target nodules was successfully performed in all patients from both groups.

**Conclusion:**

Both CT-guided MBL and CL can be readily and safely utilized for preoperative localization in individuals who had MPNs, with MBL being correlated with a shorter localization duration compared with CL.

## Introduction

Computed tomography (CT) is routinely used to screen for early-stage lung cancer, as such screening efforts have been associated with a ~ 20% reduction in lung cancer-related mortality rates [1]. On CT scans, early-stage lung tumors often appear as pulmonary nodules (PNs) [2–4]. The early diagnosis and treatment of such PNs is routinely conducted via video-assisted thoracoscopic surgery (VATS) [5–7]. However, prior studies have reported that up to 63% of patients undergoing VATS to resect PNs < 10 mm in diameter or > 5 mm from the pleura ultimately undergo conversion to thoracotomy [8]. In an effort to lower these conversion rates, many studies have thus explored the use of preoperative localization techniques to guide VATS-based sublobar (wedge or segmental) PN resection [5–7].

An estimated 17.7–22.2% of patients with PNs harbor multiple PNs (MPNs) that warrant resection [9, 10], and the simultaneous localization of these MPNs has been explained to be correlated with substantial VATS sublobar resection rates for all target nodules [9–11]. Approaches most frequently employed for localization include the use of coils, hook-wire, and methylene blue (MB) [11], with all three of these materials being associated with high (> 90%) and comparable rates of technical success when used for localization [11]. However, complication rates are generally higher for individuals undergoing hook-wire localization, with adverse events occurring in up to 90% of MPN patients undergoing hook-wire localization [12]. In contrast, MB localization (MBL) and coil localization (CL) are correlated with substantially lower rates of complication (13–55%) for patients with MPNs [11]. The relative clinical efficacy of MBL and CL techniques in patients with MPNs, however, has yet to be rigorously evaluated.

Here, we aimed to directly compare the clinical efficacy and safety of MB- and coil-based pathways to preoperative MPN localization.

## Methods

This retrospective investigation was confirmed through the Hospital Research Ethics Committee, with the need for written patient consent having been waived.

### Study design

Between January 2015 and December 2020, 137 cases with PNs underwent preoperative CT-guided localization and VATS resection in our hospital. From these cases, 31 (22.6%) underwent localization processes for MPNs. Among these patients, 15 underwent MBL between January 2015 and December 2016, while 16 underwent CL between January 2017 and December 2020. All localization procedures were performed under CT guidance.

Patients eligible for inclusion were those meeting the following criteria: (a) patients with MPNs; (b) each PN was ≥ 4 mm in diameter; (c) each PN was considered to exhibit an intermediate-to-high risk of malignancy on the basis of the clinical-radiological achievements [13]. Cases were not included if they exhibited: (a) typical diffused metastatic MPNs; (b) calcified PNs; (c) PNs that exhibited a reduction in diameter during the follow-up period; and (d) individuals with serious coagulation disorders, active infections, and/or active bleeding.

### Preoperative assessments

A chest CT examination (thickness: 1.0–1.25 mm) was used to detect PNs, which were defined as isolated round lesions ≤ 3 cm in the lungs that were not related to atelectasis, pleural effusion, or mediastinal lymphadenopathy [14]. The longest transverse diameter was used to calculate the diameter of each PN. Other preoperative parameters were also recorded, including tumor history, serum neuron-specific enolase, carcinoembryonic antigen, squamous cell carcinoma antigen, and Cyfra21-1 levels.

### MBL procedures

Before the localization procedure, we analyzed the patients’ chest CT images and determined the optimal puncture needle pathway for each target PN.

CT guidance was employed to perform all MBL processes under local anesthesia. The CT device was a Brilliance 16 CT (Philips, Cleveland, OH, USA). The tube voltage, tube current, and scanning thickness were 120 kV, 150 mAs, and 1 mm, respectively. Cases were located in an appropriate position based on the position of the target PNs. An appropriate puncture point on a patient’s skin was marked to get the shortest needle entry route meanwhile avoiding the inclusion of bullae and vessels structures, following which the puncture point was punctured with a 22G needle (Cook, IN, USA) based on the direction of the target PN. After confirming the direction of the needle tip by the repeat CT scan, further insertion into the normal pulmonary parenchyma around the target PN (within 1 cm) was carried out.. The third CT scan was performed to confirm the final position of the needle tip before injection of MB. Then, 0.1–0.3 ml of MB was injected while smoothly eliminating the needle in a way that MB remained present on the visceral pleura. After the first localization, the patient was kept in the same position or repositioned according to the location of the next target PN. The remaining PNs were localized by the same method. A one-stage CT-guided procedure was used to localize all PNs, and a repeat CT scan was then executed for the detection of any potential procedure-associated complications.

### CL procedures

The preoperative preparation was same as that in the MBL group. CT guidance was used to perform all CL procedures under local anesthesia. The CT device and parameters were same as those in the MBL group. Needle placement was performed in a manner identical to that used for MBL-based approaches, although a larger 18G needle (Precisa, Roma, Italy) was instead utilized. Subsequently, a coil (diameter: 0.038 inches, length: 50 mm; Cook) was partially pushed into the parenchyma of the lung using the needle stylet. The needle was then cautiously eliminated in a smooth motion in a way that the coil tail kept visible above the visceral pleura. After the first localization, the patient was kept in the same position or repositioned according to the location of the next target PN. The remaining PNs were localized by the same method. A one-stage CT-guided procedure was used to localize all PNs, and a repeat CT scan was then exerted for the detection of any potential procedure-associated complications.

### VATS procedure

VATS was generally performed within 3 h following MBL owing to the liquid characteristics of MB, whereas in the CL group VATS was executed within 24 h post-localization.

The visualization of the MB or coil tail above the visceral pleura was used to guide VATS sublobar resection procedures. A wedge-based approach was the standard resection technique, with segmental resection instead being performed when VATS visualization was inadequate to guarantee sufficient surgical margins. The edge of the resected tissue was at least 2 cm from the MB or coil. A one-stage VATS resection procedure was conducted for all target PNs in all patients.

Resected samples of lung parenchymal tissue were sent to our Department of Pathology for rapid pathological assessment. When nodules were diagnosed as being benign, adenocarcinoma in situ (AIS), minimally invasive adenocarcinoma (MIA), or metastatic lesions, no further resection was exerted. If nodules were instead diagnosed as invasive adenocarcinomas (IAs), additional lobectomy and systematic lymph node dissection were executed. When patients exhibited multiple IAs in different lobes of the lung, lobectomy was conducted for the most advanced PN.

### Patient assessment

Data pertaining to localization, VATS outcomes, and localization-associated complications were compared between groups. Technical achievement was defined based upon the visualization of MB or of the coil tail, as appropriate, during the VATS procedure [15], with the successful localization of all target PNs being used to define technical success on a per-patient basis [15]. The pain evaluation was made by assessing the visual analogue scale (VAS) [16]. The VAS ranged from 0 (no pain) to 10 (pain as bad as it could be) [16]. Successful sublobar resection was defined by the presence of the target PN within the resected segment or wedge of the lung parenchyma [16]. Lung hemorrhage was defined based on the detection of new-onset consolidative or ground-glass opacity proximal to the needle tract on CT [17]. Severe hemorrhage was explained by a > 2 cm width of needle tract hemorrhage on CT [17].

### Statistical analyses

SPSS 16.0 (SPSS, IL, USA) was used for all statistical analyses. Quantitative outcomes are presented as median and were scrutinized through Mann–Whitney U tests, while all other data were presented as N (%) and scrutinized through χ^2^ tests. *P* < 0.05 was the threshold of significance for this study.

## Results

### Patients

In total, this study enrolled 15 patients with 32 PNs in the MBL group and 16 patients with 36 PNs in the CL group (Table 1). Baseline data were comparable between patients in these two groups. Baseline PN-related data were comparable in both patient groups (Table 2).

**Table 1:** Comparison of patients’ baseline data between 2 groups

	Methylene blue group	Coil group	P value
Patients number	15	16	-
Age (years)	61 (Q1: 50; Q3: 68)	55 (Q1: 48; Q3: 64)	0.489
Gender			0.576
Male	6	8	
Female	9	8	
Previous tumor history	1	2	1.000
Emphysema	1	0	0.484
Tumor marker levels			
Neuronspecific enolase (U/ml)	12.3 (Q1:11.6; Q3: 13.2)	12.1 (Q1:10.7; Q3: 13.7)	0.58
Carcinoembryonicantigen (ng/ml)	1.9 (Q1:1.6; Q3: 3.2)	1.5 (Q1:1.3; Q3: 2.3)	0.205
Squamouse cell carcinoma antigen (μg/L)	1 (Q1:0.8; Q3: 1.6)	1.2 (Q1:0.6; Q3: 1.6)	0.751
Cyfra21-1 (ng/ml)	2.1 (Q1:1.5; Q3: 2.6)	1.6 (Q1:1.2; Q3: 2.6)	0.372

**Table 2:** Comparison of pulmonary nodules’ baseline data between 2 groups

	Methylene blue group	Coil group	P value
Pulmonary nodules number	32	36	1.000
Patients with 2 nodules	13	13	
Patients with ≥ 3 nodules	2	3	
Natures of the nodules			0.236
Solid	10	14	
Mixed GGN	2	6	
Pure GGN	20	16	
Nodules diameter (mm)	6 (Q1: 4.3; Q3: 9.8)	6 (Q1: 4; Q3: 9)	0.509
Nodule-pleura distant (mm)	5 (Q1: 4; Q3: 10)	4 (Q1: 1; Q3: 9)	0.07
≤ 10 mm	21	29	0.164
> 10 mm	11	7	
Sites of the multiple pulmonary nodules			0.124
Unilateral multiple pulmonary nodules	15	12	
Bilateral multiple pulmonary nodules	0	4	
Lung sides			0.506
Left	15	14	
Right	17	22	
Lung lobes			0.230
Upper	18	15	
Non-upper	14	21	

*GGN* ground glass nodule

## CT-guided localization outcomes

The PN- and patient-based technical success rates were both 100% in the MBL group, whereas in the CL group these respective rates were 97.2% (35/36) and 93.8% (15/16). The coil did not become dislodged in any patients. Technical failure occurred for one PN in the CL group as the coil was entirely embedded into the lung parenchyma. The rates of technical achievement did not vary substantially between the MBL and CL groups (Table 3). The median duration of CT-guided localization was remarkably shorter in the MBL group relative to the CL group (18 min vs. 29.5 min, *P* < 0.001).

**Table 3:** Localization-related results

	Methylene blue group	Coil group	P value
Technical success			
Based on nodules	100% (32/32)	97.2% (35/36)	1.000
Based on patients	100% (15/15)	93.8% (15/16)	1.000
Post-localization VAS score	3 (Q1: 2; Q3: 3)	3 (Q1: 2; Q3: 3)	0.758
Duration of localization procedures (min)	18 (Q1: 17; Q3: 23)	29.5 (Q1: 26.3; Q3: 38.8)	< 0.001
Complications			
Pneumothorax	20.0% (3/15)	18.8% (3/16)	1.000
Lung hemorrhage	20.0% (3/15)	25% (4/16)	1.000
Severe hemorrhage	0%	0%	-

*VAS* visual analogue scale

Rates of pneumothorax (20.0% vs. 18.8%, *P* = 1.000) and lung hemorrhage (20.0% vs. 25.0%, *P* = 1.000) were comparable in these two groups, and no patients experienced any instances of severe hemorrhage. In no case did the observed complications result in the delay of the VATS procedure. The median post-localization VAS were 3 and 3 in the MBL and CL groups, respectively (*P* = 0.758).

### VATS procedure outcomes

In the MBL and CL groups, the median interval between localization and VATS was 1 h and 15.5 h, respectively (*P* < 0.001). Rates of sublobar resection technical success were 100% in both the MBL and CL groups, with all target PNs having been successfully resected via a one-stage VATs approach. Despite the technical failure of CL for one PN, this nodule was successfully removed via wedge resection through the palpation of the intra-pulmonary coil. Details regarding the surgical approach and final diagnostic outcomes are compiled in Table 4. Ultimately, 2 and 1 cases in the MBL and CL groups, accordingly, underwent lobectomy owing to the diagnosis of IA nodules. Six and 3 patients in the MBL and CL groups had the multiple lung cancers. Surgical type and VATS duration did not vary notably between the MBL and CL groups (*P* = 0.433 and 0.092, respectively).

**Table 4:** Surgical types and final diagnoses

	Methylene blue group	Coil group	P value
Technical success of wedge/segmental resection	100%	100%	–
Time from localization to surgery (h)	1 (Q1: 1; Q3: 1.5)	15.5 (Q1: 5; Q3: 17.5)	< 0.001
Duration of VATS (min)	90 (Q1: 60; Q3: 165)	155 (Q1: 82.5; Q3: 203.8)	0.092
Types of resection			0.433
Wedge resection	24	30	
Segmental resection	6	5	
Wedge resection + lobectomy	3	1	
Final diagnosis			0.023
Invasive adenocarcinoma	6	2	
MIA	4	1	
AIS	8	7	
Atypical hyperplasia	1	10	
Benign	13	16	
Patients with multiple lung cancers	6	3	0.193

*VATS* video-assisted thoracoscopic surgery, *MIA* mini-invasive adenocarcinoma, *AIS* adenocarcinoma in situ

## Discussion

Preoperative CT-guided localization is frequently used to prepare patients scheduled to undergo VATS sublobar resection for peripheral PNs, and such localization is associated with lower rates of VATS anatomic resection or thoracotomy [18]. Relative to the localization of a single PN, the MB- or coil-based localization of MPNs is associated with similar comfort, safety, and reliability [9, 19].

Here, we assessed the safety and efficacy outcomes associated with the MB- and coil-based preoperative localization of MPNs. Technical success rates were comparable for both of these procedures at both the per-PN (100% vs. 97.2%) and per-patient (100% vs. 93.8%) levels. These high success rates indicate that both MB and coils can be effectively used for the localization of MPNs. In addition, we found the MBL approach to be correlated with shorter localization progress duration compared with the CL approach (18 min vs. 29.5 min, *P* < 0.001). This suggests that the MBL approach can be more quickly executed relative to the CL procedure, which demands a greater level of technical skill to avoid the accidental insertion of the entirety of the coil [5, 6, 15].

MB overflow is the major reason of the technical failure of MB localization. In this study, we only injected 0.1–0.3 ml of MB for each PN and the MB was injected smoothly. Some researchers considered that an excessive MB volume can contribute to MB overflow [20]. Furthermore, fast injection also can cause the MB overflow [20].

We observed 100% technical success rates for VATS sublobar resection in both the MBL and CL groups in this study, with all procedures having been successfully performed via a one-stage approach. Roughly 20% of patients with MPNs harbor multiple early-stage lung cancers [15], and these patients are likely to benefit from multiple VATS resection procedures [21, 22]. However, one-stage resection may be superior to two-stage resection with respect to the risk of disease progression [23].

While MBL is simpler than CL with respect to the level of technical skill required, there are certain advantages to CL over MBL. Notably, VATS must be conducted as quickly as possible following MBL owing to the fact that MB is a liquid, whereas the interval between localization and VATS can be extended for CL as the coil is solid and not at risk of diffusion or signal loss in cases where VATS must be delayed.

In our patient cohort, we found that rates of pneumothorax (20.0% vs. 18.8%, *P* = 1.000) and lung hemorrhage (20.0% vs. 25.0%, *P* = 1.000) were comparable in both groups, explaining that these two localization strategies can exhibit similar safety profiles. While different needle sizes were used in the MBL and CL groups (22G vs. 18G), prior evidence suggests that pneumothorax rates do not rise significantly provided the size of the utilized needle remains under 16G [24]. Importantly, the pneumothorax rates in both the MBL and CL groups were relatively low (20.0% and 18.8%, respectively), with these rates being substantially lower than those reported for hook-wire localization (mean: 56%) [11].

Coil is a metal localization material and dislodgment is a key complication. Dislodgment can cause technical failure of localization and pneumothorax [25, 26]. When performing the coil localization, partial coil is inserted into the lung parenchyma and the coil tail may extend into the chest wall, leaving it susceptible to dislodgement in response to respiratory movement [25]. However, the coil dislodgement rate was significantly lower than hook-wire dislodgement rate [27].

There are some limitations to this study. First, this was a retrospective study and it is therefore susceptible to the potential for bias. Second, patients in the included groups were processed during various periods of time. Even so, the baseline data for patients in these two groups were comparable, potentially lowering the risk of selection bias. Third, although the baseline data were comparable between 2 groups, no patient in MBL group had bilateral MPNs. This finding also might cause the selective bias. Fourth, the selected sample size was limited, underscoring the need for additional prospective randomized trials with larger sample sizes to validate these findings.

## Conclusion

In summary, we herein found that both CT-guided MBL and CL can be safely and effectively utilized for preoperative MPN localization, providing appropriate guidance for subsequent one-stage VATS sublobar resection. Of these two approaches, MBL was correlated with a shorter localization.

## Data Availability

The data that support the findings of this study are available from the corresponding author upon reasonable request.

## Abbreviations

CL: Coil localization
CT: Computed tomography
MB: Methylene blue
MBL: Methylene blue localization
MPN: Multiple pulmonary nodule
PN: Pulmonary nodule
VAS: Visual analogue scale
VATS: Video-assisted thoracoscopic surgery
